# Machine learning-based cognitive load prediction model for AR-HUD to improve OSH of professional drivers

**DOI:** 10.3389/fpubh.2023.1195961

**Published:** 2023-08-03

**Authors:** Jian Teng, Fucheng Wan, Yiquan Kong, Ju-Kyoung Kim

**Affiliations:** ^1^School of Mechanical and Electrical Engineering, Lingnan Normal University, Zhanjiang, China; ^2^College of Education, Sehan University, Yeongam, Jeollanam-do, Republic of Korea; ^3^College of Computer and Intelligent Manufacturing, Lingnan Normal University, Zhanjiang, China

**Keywords:** AR-HUD interface design, OSH, cognitive load, machine learning, IVPM-GA

## Abstract

**Motivation:**

Augmented reality head-up display (AR-HUD) interface design takes on critical significance in enhancing driving safety and user experience among professional drivers. However, optimizing the above-mentioned interfaces poses challenges, innovative methods are urgently required to enhance performance and reduce cognitive load.

**Description:**

A novel method was proposed, combining the IVPM method with a GA to optimize AR-HUD interfaces. Leveraging machine learning, the IVPM-GA method was adopted to predict cognitive load and iteratively optimize the interface design.

**Results:**

Experimental results confirmed the superiority of IVPM-GA over the conventional BP-GA method. Optimized AR-HUD interfaces using IVPM-GA significantly enhanced the driving performance, and user experience was enhanced since 80% of participants rated the IVPM-GA interface as visually comfortable and less distracting.

**Conclusion:**

In this study, an innovative method was presented to optimize AR-HUD interfaces by integrating IVPM with a GA. IVPM-GA effectively reduced cognitive load, enhanced driving performance, and improved user experience for professional drivers. The above-described findings stress the significance of using machine learning and optimization techniques in AR-HUD interface design, with the aim of enhancing driver safety and occupational health. The study confirmed the practical implications of machine learning optimization algorithms for designing AR-HUD interfaces with reduced cognitive load and improved occupational safety and health (OSH) for professional drivers.

## 1. Introduction

### 1.1. Background and significance

AR-HUD technology has become increasingly popular in the transportation industry over the past few years as an advanced driver assistance technology that is capable of improving OSH for professional drivers (1). AR-HUD technology is promising in providing drivers with critical information while minimizing visual distraction, improving safety and reducing cognitive load, which are recognized as vital factors for OSH (2). AR-HUD technology offers several advantages for professional drivers, which covers real-time information that takes on critical significance in drivers to perform their jobs safely and efficiently (e.g., speed, fuel levels, and engine temperature for commercial truck drivers) (3). Furthermore, AR-HUD technology shortens the time that drivers have to take their eyes off the road, such that OSH can be improved by minimizing visual distractions (4). Moreover, AR-HUD technology can help reduce cognitive load by providing drivers with only the necessary information, such that they are enabled to focus on their primary task of driving (5).

However, existing AR-HUD designs may not be optimal for professional drivers who are dependent on more specialized and customized information to perform their jobs effectively (6). Consequently, OSH risks may be posed since the information presented on the display may not be easily customizable, making it difficult for professional drivers to receive the specialized information they require (7–9). Furthermore, the display is likely to increase cognitive load, particularly for drivers not accustomed to using AR-HUD technology, such that fatigue and other OSH issues can be triggered (10–12). Accordingly, designing AR-HUD interfaces that are tailored to the specific needs of professional drivers takes on critical significance in improving OSH.

The significance of this study to occupational health and safety (OHS) is indicated in its potential in strengthening safety measures and improving professional drivers’ quality of life. The study leverages machine learning and optimization techniques to design Augmented Reality Heads-Up Displays (AR-HUDs), which can present more insights into improving driving tasks’ ergonomic aspects, such that accident risks can be mitigated, and driver well-being can be elevated. Notably, the optimization of AR-HUDs with the IVPM-GA method is conducive to reducing cognitive load, which when excessive, can lead to driver fatigue and higher accident rates. Furthermore, the IVPM method’s prowess in localizing eye-tracking hotspots and predicting extreme points on graphs is capable of enhancing AR-HUDs’ visual ergonomics, minimizing eye strain, and preventing health issues (e.g., headaches or vision issues). Additionally, a well-optimized AR-HUD interface can enhance the overall user experience, reducing stress and discomfort during extended operation periods, which contributes to mental health benefits. The study’s findings can guide subsequent OHS policies and training programs, such that an evidence-based method can be provided to interface design that can be incorporated into professional drivers’ training. The optimized AR-HUD interface is promising in integrating with existing driver assistance systems, such that timely and relevant information can be presented, and cognitive load reduction and overall driving safety enhancement can be facilitated.

### 1.2. Research items

The item of this study refers to optimizing AR-HUD visual interaction for professional drivers using machine learning techniques to reduce visual fatigue and cognitive load. To be specific, this study aimed at developing an optimized AR-HUD interface design using a genetic algorithm based on an Image Viewpoint Prediction Model (IVPM), comparing the effectiveness of the IVPM method and the conventional Backpropagation Genetic Algorithm (BP-GA) method in optimizing AR-HUD interface design, assessing the implications of optimized AR-HUD interfaces for the occupational health and safety (OHS) of professional drivers, employing machine learning to predict cognitive load in AR-HUD design and assess its impact on driving performance and user experience, and laying a solid basis for future Occupational Safety and Health (OSH) policies and training programs following the findings of this study. The above-mentioned research items can guide the exploration of AR-HUD interface design optimization, the comparison of different optimization methods, and the implications of the above-described methods for OHS in professional driving.

The rest of this study is organized as follows. In Section 2, a comprehensive review of related research on AR-HUD interfaces, cognitive load, and optimization methods is presented. In Section 3, the methodology applied in this study is introduced, including the IVPM method and the GA optimization algorithm. In Section 4, the experimental setup, data collection, and the assessment of the proposed IVPM-GA method are presented, compared with the conventional BP-GA method. In Section 5, the interpretation of the results and the implications of the findings are discussed, and the limitations of the study are acknowledged. Lastly, in Section 6, the research findings are summarized, the innovation of the IVPM-GA method is emphasized, and future research directions in the field of AR-HUD technology and interface optimization are proposed.

## 2. Literature review

### 2.1. AR-HUD technology in assisted driving

The research on cognitive load of drivers using an AR-HUD in assisted driving refers to a multidisciplinary field dedicated to designing and assessing interactive systems to enhance the driver experience while improving occupational safety and health (6). AR-HUD technologies have aroused significant attention in the HCI field over the past few years. AR refers to a technology that superimposes computer-generated virtual objects onto the real world, creating a mixed reality environment where the virtual and physical worlds coexist HUD (12). Besides, it is a display technology that projects information onto a transparent screen or a windshield, such that users are enbaled to view information without looking away from the road or the task at hand (13). AR-HUD technology combines the benefits of both AR and HUD to provide a more intuitive and immersive user experience. It has been extensively employed in a wide variety of domains (e.g., automotive, aviation, and military) for tasks (e.g., navigation, communication, and training) (14) nEVERTHELESS, designing effective AR-HUD interfaces remains a challenge due to the complexity of the technology and the need to balance the visual and cognitive demands of the user (15).

The design of AR-HUD interfaces poses several challenges for HCI (Human-Computer Interaction) designers: the design should consider the user’s cognitive load, as too much information presented on the display can overwhelm the user and lead to decreased performance and safety issues (16). Furthermore, the interface should be developed to maximize the user’s attention and minimize distraction while providing relevant and timely information (17).

### 2.2. Machine learning in AR-HUD design to improve OSH of drivers

Machine learning (ML) and deep learning (DL) have been confirmed as subfields of artificial intelligence that are highly promising in facilitating the design and assessment of interactive systems in HCI. ML refers to a method of teaching computers to learn patterns from data without being explicitly programmed (18). DL refers to a subset of ML that uses artificial neural networks to learn complex patterns from large datasets (19).

In the research of AR-HUD human-computer interaction, ML and DL have been employed for tasks (e.g., user modeling, gesture recognition, emotion detection, as well as speech recognition) (20). Besides, they have been adopted to optimize the design of interfaces by predicting user behavior and preferences, reducing cognitive load, and improving usability (21).

The research on the optimization of AR-HUD interface design using machine learning in recent years is presented in the following: Optimization of AR-HUD interface design by machine learning. Conati et al. (22) using machine learning research methods, the use of interaction data was explored in this study as an alternative source to predict cognitive abilities during visualization processing when eye-tracking data was not available, and the accuracy of user models was assessed based on different data sources and modalities. Results indicated the potential for using interaction data to enable adaptation for interactive visualizations, and the value of combining multiple modalities for predicting cognitive abilities. Besides, the effect of noise in gaze data on prediction accuracy was also examined. In Oppelt et al.’s study, (23), machine learning algorithms were trained and assessed using single and multimodal inputs to distinguish cognitive load levels. The model behavior was carefully assessed, and feature importance was investigated. A novel cognitive load test was introduced, and a cognitive load database was generated. Variations were validated using statistical tests, and novel classification and regression tasks were introduced for machine learning (12). Becerra-Sánchez et al. (24) uses n-back task as an auxiliary task to induce the cognitive load of the main task (i.e., driving) in three different driving simulation scenarios. Multi-modal machine learning method is used to classify drivers’ cognitive load. Multi-component signals, i.e., physiological measurement and vehicle characteristics, are used to overcome the noise and mixed factors in physiological measurement. Feature selection algorithm is used to identify the optimal feature set, and random forest algorithm shows better performance than other algorithms. It is found that using multi-component feature classifier can classify better than using features from a single source. In the research of Jacobé de Naurois et al. (25) by analyzing physiological and behavioral indicators (e.g., heart rate, blink duration and driving behavior), machine learning method was employed to detect and predict drivers’ drowsiness. AS indicated by the result, increasing information (e.g., driving time and participant information) can increase the accuracy of drowsiness detection and prediction. The optimal performance was achieved through the combination of behavioral indicators and additional information. The developed model is capable of detecting the drowsiness level with a mean square error of 0.22, and carrying out prediction when it will reach a given drowsiness level with a mean square error of 4.18 min.

The following is the research on the optimization of AR-HUD interface design using deep learning over the past few years. Kang et al. (26) proposed a novel deep learning-based hand interface for immersive virtual reality, providing realistic interactions and a gesture-to-action interface without the need for a graphical user interface. An application was developed to compare the proposed interface with existing GUIs, and a survey experiment assessed its positive effects on user satisfaction and sense of presence. Zhou (27) investigated the technical challenges in applying AR-HUD systems in practical driving scenarios. A lightweight deep learning-based object detection algorithm was proposed, while a system calibration method and a wide variety of image distortion correction techniques were developed. The methods were integrated into a multi-eye AR control module for the AR-HUD system, which was assessed through road experiments. The results confirmed the effectiveness of the proposed methods in enhancing driving safety and user experience. Rahman et al. (28) employed deep learning to develop a vision-based method, with the aim of classifying a driver’s cognitive load to improve road safety. In the study, non-contact solutions were investigated through image processing, with a focus on eye movements. Five machine learning models and three deep learning architectures were developed and tested, achieving up to 92% classification accuracy. This non-contact technology is promising in contributing to advanced driver assistive systems. Methuku (29) proposed the use of a deep learning system based on Convolutional Neural Network (CNN) to classify in-car driver responses and create an alert system to mitigate vehicle accidents. The system was developed using transfer learning with ResNet50 and achieved an accuracy of 89.71%. The study provided key conclusions and discussed the significance of the research in practical applications.

### 2.3. Genetic algorithm in AR-HUD design to improve OSH of drivers

Optimization techniques (e.g., genetic algorithms (GA), particle swarm optimization (PSO), and simulated annealing (SA)) have been extensively employed in HCI to optimize the design and assessment of interactive systems. The above-mentioned techniques were adopted to find the optimal solution from a large set of possible solutions by iteratively assessing and modifying the design variables (30). GA is a search algorithm that mimics the process of natural selection to find the optimal solution to a problem. It starts with a set of random solutions and iteratively improves them by applying genetic operations such as selection, crossover, and mutation (31). PSO is a swarm-based optimization technique simulating the social behavior of a group of individuals to determine the optimal solution (32). SA refers to a probabilistic optimization technique simulating the process of cooling a material to determine the minimum energy state (33). Goli et al. (34) developed a complex model for cell formation in a manufacturing system using automated guided vehicles (AGVs). It introduced a hybrid genetic algorithm and a whale optimization algorithm to address the problem. As revealed by the results, the above-described algorithms outperform existing solutions in efficiency and accuracy, with the whale optimization algorithm proving to be optimal. Tirkolaee and Aydin (35) introduced a fuzzy bi-level Decision Support System (DSS) for optimizing a sustainable supply chain for perishable products. It employs a hybrid solution technique based on possibilistic linear programming and Fuzzy Weighted Goal Programming (FWGP) to cope with uncertainty and ensure sustainability. The proposed methodology outperforms existing methods, solving a problem with over 2.2 million variables and 1.3 million constraints in under 20 min. The above-mentioned studies highlight the effectiveness of optimization techniques in solving complex issues. The use of hybrid algorithms and the application of the above-described techniques to practical issues underscore their practical relevance. In this study, the above-mentioned findings suggest that similar methods could enhance the effectiveness of our IVPM-GA method for AR-HUD interface design, potentially leading to significant improvements in Occupational Safety and Health (OSH) for professional drivers.

As revealed by the literature review, the backpropagation genetic algorithm (BP-GA) has been extensively employed to predict cognitive load based on eye-tracking data, such that AR-HUD and OSH of professional drivers can be better optimized (36, 37). The significance of BP-GA lies in its ability to optimize the AR-HUD interface design while considering user cognitive load (36), such that the OSH of professional drivers can be ultimately enhanced. By optimizing interface design, BP-GA reduces cognitive load, improves user experience, and ultimately lowers the risk of accidents caused by distraction. However, a potential drawback of BP-GA is that it is likely to converge to local optima instead of global optima (38). To address the above-mentioned issue, the IVPM-GA method was introduced in this study, employing the lightweight deep learning image viewpoint prediction model (IVPM) algorithm to predict cognitive load, as well as a vision-based cognitive load prediction method based on machine learning. This method is capable of overcoming the limitations of existing research that relied solely on eye-tracking data, which may not fully encompass cognitive abilities. The IVPM-GA method provides more comprehensive and accurate predictions of cognitive load and can be adopted to optimize AR-HUD interface design in depth.

The main novelties of this study lie in the application of advanced machine learning techniques, specifically the Image Viewpoint Prediction Model (IVPM) and the Backpropagation Genetic Algorithm (BP-GA), to optimize the design of Augmented Reality Head-Up Display (AR-HUD) interfaces for professional drivers. This study is unique in its method to reducing visual fatigue and cognitive load, key factors that can impact the occupational health and safety (OHS) of professional drivers. By leveraging machine learning to predict cognitive load in AR-HUD design, this study offers a novel way to enhance driving performance and user experience.

This study represents a contribution to the field of human-computer interaction, demonstrating how advanced technologies such as AR-HUD can be effectively adopted to enhance driver safety and occupational health. Thus, this study advocates for the development of safer and more ergonomic professional driving environments, a requisite consideration in the modern fast-paced world, substantiating its relevance to OSH.

## 3. Materials and methods

### 3.1. Experiment and dataset collection

#### 3.1.1. Experimental environment and equipment

**Figure 1:**
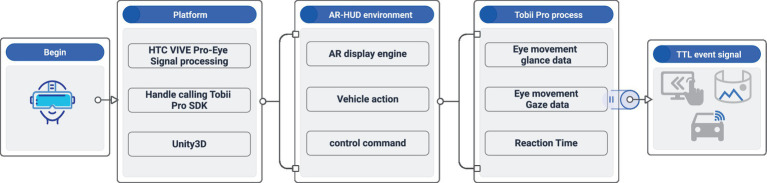
Structure of virtual reality driving assistant test system.

In the experiment, a self-designed integrated helmet with eye tracking and AR function was adopted to provide 360 immersive driving simulation and eye tracking experiments. The operating equipment refers to a high-performance desktop computer, and the steering wheel, pedals, and gears were connected to the notebook computer through relevant ports. To enable the test subjects to acquire prominent real driving experience and interactive experience, Unity3D engine was employed in the experiment to design and build a driving assistance test system on AR platform. The system compiled the logic codes of vehicle actions (e.g., vehicle acceleration, maximum speed, and deceleration when braking). Moreover, Tobii Pro adaptive eye movement analysis function was loaded in Unity3D environment. The eye movement analysis SDK exhibited the capability of providing eye movement data stream signals (e.g., the gaze time of left and right eyes as the original data), displaying the gaze origin (3D eye coordinates), gaze point and space comfort distance L(GazeData), and synchronizing the external TTL event signals of the input port, with the aim of synchronizing eye movement data and other biometric data streams. Figure 1 illustrates the system architecture.

The participants were a group of 40 professional drivers with normal or corrected vision. The group had a mean age of 31.36 years (standard deviation = 4.97). At the time of testing, the participants were in good mental condition and none of them experienced VR simulation sickness. They all held a driver’s license and had an average driving experience of over 5 years.

**Figure 2:**
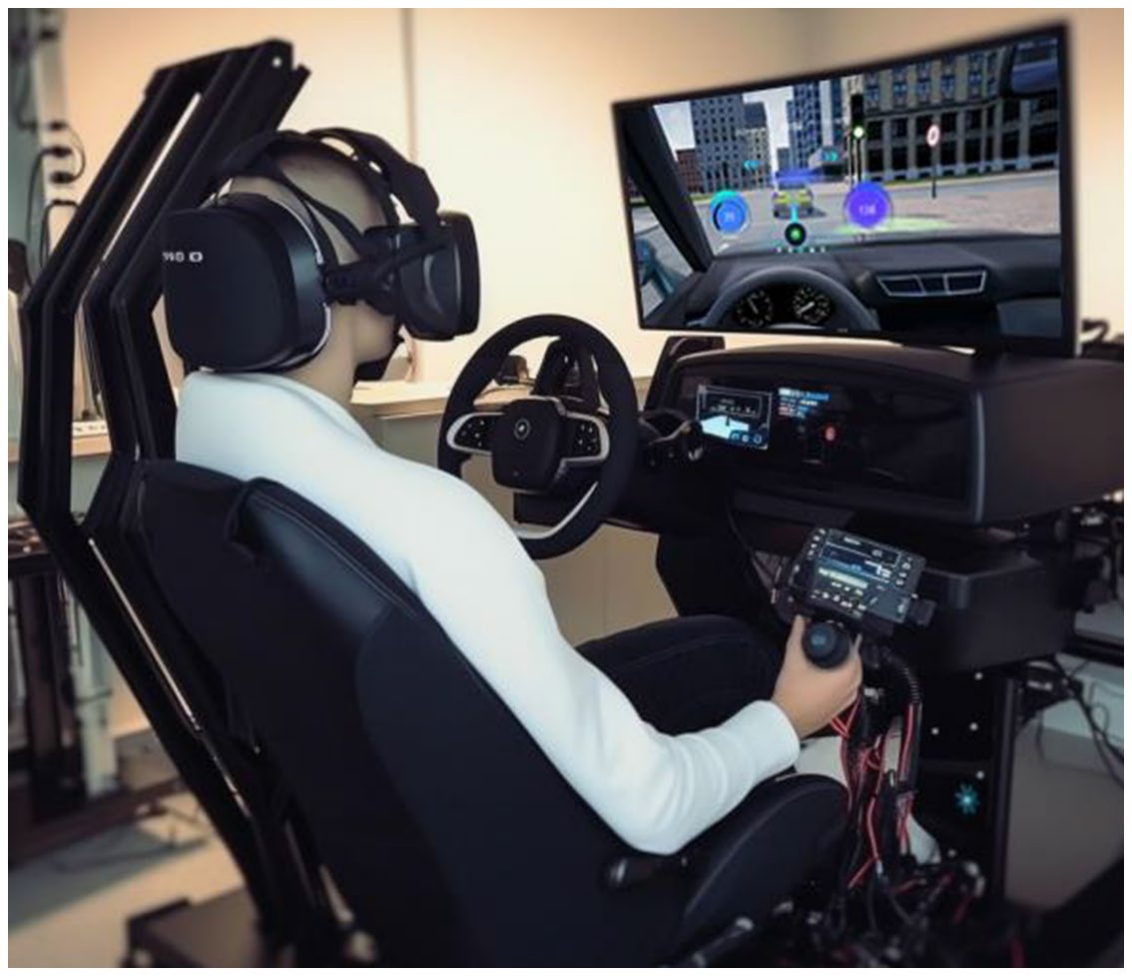
Experimental environment and equipment.

The simulation platform was illustrated in Figure 2, comprised of a mock car cockpit, large screen, audio-visual components, and an AR-HUD helmet with eye-tracking capabilities. Unity3D software provides panoramic modeling of road conditions and real-time data recording. The helmet, integral to this study, houses an Eye Tracking Module sensor and Eye Tobii VR lens on the forehead, capturing eye movements during AR-HUD display. The above-described signals are wirelessly transmitted to Tobii Pro Lab software for analysis.

#### 3.1.2. Experimental interface design of AR-HUD display

**Figure 3:**
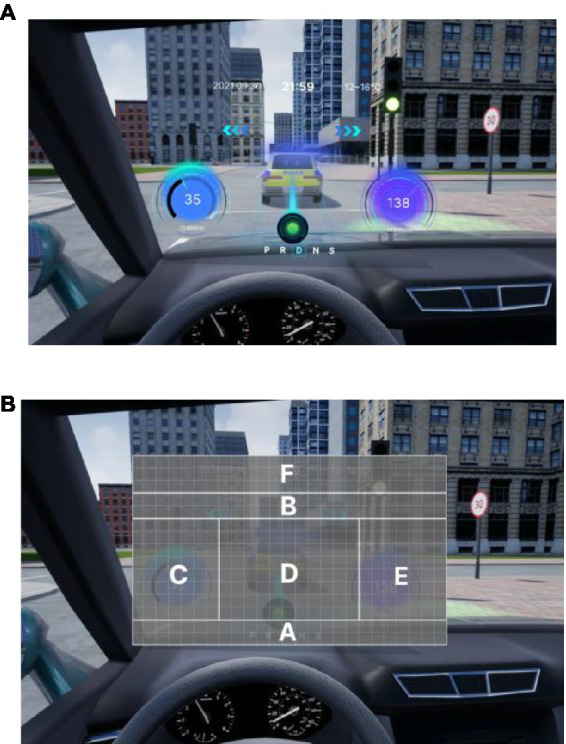
AR-HUD design scheme: **(A)** AR-HUD interface design; **(B)** AR-HUD interface layout.

According to the design principles of AR-HUD and the visual characteristics of the human eye, AR-HUD is located in the lower left corner of the windshield, which is also the driver’s central visual area, as shown in Figure 3A. For vehicles at a speed less than 75 km/h, the visual information of AR-HUD fell into 85° of binocular vision. For vehicles at a speed between 75 and 100 km/h, the information was less than 65% of binocular vision. For vehicles at a speed exceeding 100 km/h, the information fell into 40° of binocular vision. As depicted in Figure 3B, the AR-HUD interface fell into six areas as follows: A: driving status information and gear status; B: navigation information, including navigation instructions and other driving prompts; C: speed information; D: warning information area, including pedestrian warning, frontal collision warning, side collision warning, driver abnormality warning, road speed limit warning, and lane departure warning; E: default display of speed information, and the driver can customize other display information; F: basic driving information area, displaying time and date information. To explore the relationship between the interface design of AR-HUD design elements, the layout of the A-F components and the design of the subcomponents will be presented in subsequent experiments.

#### 3.1.3. Experimental method

This study delineates AR-HUD information design patterns and tests varied visual colors, layouts, and components. Each driver executes 6 tasks involving random layout and component designs, with a 10-min rest between tasks. This process produces 240 samples for deep learning prediction. Each driving scenario lasts 60 s, with eye movement parameters extracted in 30-s intervals.

**Figure 4:**
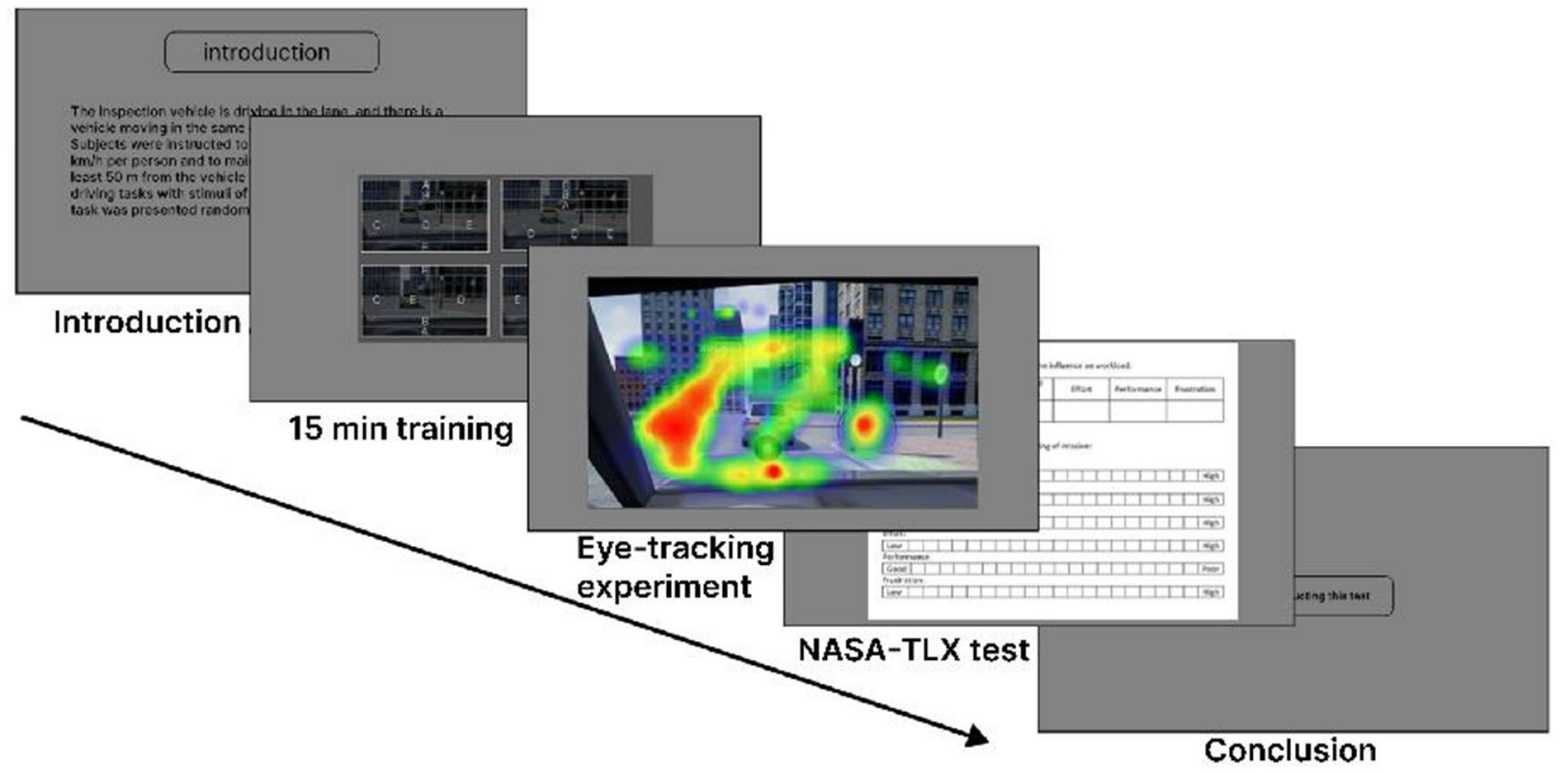
Experimental process.

Figure 4 illustrates the procedure, where a wide variety of AR-HUD visual schemes were covered, randomly presented to measure users’ visual cognitive load. The test involved driving at 50 kilometers per hour, maintaining a minimum 50-meter distance from a preceding car, as well as locating and stating information displayed on the AR-HUD. Data were collected on driving behavior and eye movement after the respective task.

The experiment was performed on city roads under good weather and moderate traffic conditions. A 15-min training familiarized participants with simulator operation, visual search, target warnings, and driving modes. After the respective task, participants employed the NASA-TLX form to assess their subjective cognitive load regarding AR-HUD use. (39–42).

### 3.2. Experimental results and analysis

Eye-tracking data segments were taken before and after visual search for 30 s. According to the Shapiro-Wilke test, the differences in the four groups of experimental data were normally distributed (*p* < 0.05), meeting the assumption of parametric testing. Accordingly, using a paired t-test, eye-tracking variations in drivers were tested through visual search and target discovery. The descriptive statistics and matched t-test results for visual search are shown in Table 1. Significant variations in eye-tracking indicators with different visual layouts of the flat display show that visual search for targets can effectively bring the driver’s attention back to the control loop and make them aware of potential dangers.

**Table 1:** Statistical results of measurement indexes of eye movement experiment.

Num	Gaze/ms	Glance/ms	Reaction/ms	NASA-TLX	*P*	95% confidence interval
1	0.44	0.46	2.28	12.10	0.03	(0.92, 1.33)
2	0.58	0.65	2.38	12.60	0.02	(0.89, 1.17)
3	0.38	0.23	1.04	5.52	0.00	(0.21, 0.69)
4	0.71	0.56	0.75	4.25	0.02	(1.18, 3.50)
5	0.86	0.59	1.30	6.93	0.01	(0.95, 1.50)
6	0.81	0.58	1.83	9.71	0.05	(0.89, 1.14)
7	0.54	0.53	2.15	11.42	0.03	(0.21, 1.69)
8	0.99	0.99	2.08	11.14	0.05	(0.98, 4.50)
9	0.56	0.94	1.72	9.16	0.04	(0.95, 2.50)
10	0.21	0.37	2.47	13.12	0.81	(0.82, 1.17)
11	0.35	0.38	1.72	9.13	1.05	(0.71, 0.69)
12	0.41	0.47	2.13	11.33	0.33	(1.98, 4.50)
13	0.40	0.45	2.64	14.32	0.23	(0.95, 1.50)
14	0.54	1.05	1.28	6.82	0.05	(0.49, 1.11)
15	0.37	0.25	1.04	5.52	0.48	(0.31, 0.59)
16	0.46	0.84	1.53	8.13	0.71	(0.86,1.13)
17	0.11	0.27	2.28	12.13	0.95	(0.23, 1.39)
18	0.22	0.28	1.53	8.12	0.23	(0.94, 3.50)
19	0.31	0.37	1.94	10.32	0.13	(0.92, 1.50)
20	0.41	0.39	2.08	11.15	0.04	(0.94, 1.32)
21	0.52	0.57	1.72	9.05	0.03	(0.78, 1.21)
22	0.62	0.78	2.28	12.25	0.03	(1.12, 2.08)
23	0.72	0.89	2.47	13.35	0.03	(1.32, 2.56)
24	0.38	0.35	2.13	11.32	0.05	(0.85, 1.46)
25	0.55	0.62	1.94	10.28	0.04	(0.92, 1.35)
26	0.47	0.43	2.08	11.18	0.04	(1.05, 2.02)
27	0.66	0.79	1.53	8.11	0.02	(1.22, 2.17)
28	0.42	0.38	1.72	9.05	0.02	(0.98, 1.32)
29	0.59	0.66	1.28	6.81	0.02	(1.12, 2.09)
30	0.43	0.41	1.53	8.15	0.03	(0.89, 1.25)
31	0.57	0.61	1.94	10.25	0.04	(1.08, 2.18)
32	0.48	0.46	2.28	12.19	0.04	(0.96, 1.35)
33	0.63	0.76	1.72	9.03	0.04	(1.21, 2.09)
34	0.51	0.54	2.47	13.41	0.05	(1.05, 2.32)
35	0.69	0.85	2.13	11.29	0.03	(1.12, 2.08)
36	0.45	0.42	1.94	10.31	0.04	(0.98, 1.29)
37	0.58	0.64	1.53	8.09	0.03	(1.15, 2.06)
38	0.53	0.58	1.72	9.01	0.04	(1.08, 2.11)
39	0.61	0.74	2.28	12.17	0.05	(1.01, 2.28)
40	0.49	0.47	2.47	13.38	0.04	(0.94, 1.42)
41	0.65	0.81	2.13	11.28	0.04	(1.05, 2.12)
42	0.56	0.63	1.94	10.30	0.04	(0.99, 1.33)
43	0.47	0.44	1.53	8.10	0.05	(1.12, 2.25)
44	0.62	0.77	1.72	9.03	0.04	(1.18, 2.09)
45	0.54	0.59	2.47	13.39	0.04	(1.02, 2.22)
46	0.68	0.84	2.13	11.26	0.04	(1.08, 2.16)
47	0.49	0.48	1.94	10.33	0.05	(0.95, 1.37)
48	0.59	0.68	1.53	8.07	0.05	(1.16, 2.19)
49	0.55	0.61	1.72	9.01	0.04	(1.12, 2.11)
50	0.64	0.80	2.28	12.15	0.04	(1.05, 2.28)
51	0.62	0.78	2.28	12.25	0.03	(1.12, 2.08)
52	0.72	0.89	2.47	13.35	0.03	(1.32, 2.56)
53	0.41	0.39	2.08	11.15	0.04	(0.94, 1.32)
54	0.58	0.57	1.72	9.05	0.03	(0.78, 1.21)
55	0.51	0.54	2.47	13.41	0.05	(1.05, 2.32)
56	0.69	0.85	2.13	11.29	0.03	(1.12, 2.08)
57	0.45	0.42	1.94	10.31	0.04	(0.98, 1.29)
58	0.63	0.76	1.72	9.03	0.04	(1.21, 2.09)
59	0.54	0.59	2.47	13.39	0.04	(1.02, 2.22)
60	0.68	0.84	2.13	11.26	0.04	(1.08, 2.16)
61	0.49	0.48	1.94	10.33	0.05	(0.95, 1.37)
62	0.59	0.68	1.53	8.07	0.05	(1.16, 2.19)
63	0.55	0.61	1.72	9.01	0.04	(1.12, 2.11)
64	0.64	0.80	2.28	12.15	0.04	(1.05, 2.28)
65	0.47	0.44	1.53	8.10	0.05	(1.12, 2.25)
66	0.61	0.74	1.72	9.03	0.04	(1.18, 2.09)
67	0.56	0.63	1.94	10.30	0.04	(0.99, 1.33)
68	0.41	0.39	2.08	11.15	0.04	(0.94, 1.32)
69	0.58	0.57	1.72	9.05	0.03	(0.78, 1.21)
70	0.51	0.54	2.47	13.41	0.05	(1.05, 2.32)
71	0.69	0.85	2.13	11.29	0.03	(1.12, 2.08)
72	0.45	0.42	1.94	10.31	0.04	(0.98, 1.29)
73	0.63	0.76	1.72	9.03	0.04	(1.21, 2.09)
74	0.54	0.59	2.47	13.39	0.04	(1.02, 2.22)
75	0.68	0.84	2.13	11.26	0.04	(1.08, 2.16)
76	0.49	0.48	1.94	10.33	0.05	(0.95, 1.37)
77	0.59	0.68	1.53	8.07	0.05	(1.16, 2.19)
78	0.55	0.61	1.72	9.01	0.04	(1.12, 2.11)
79	0.64	0.80	2.28	12.15	0.04	(1.05, 2.28)
80	0.47	0.44	1.53	8.10	0.05	(1.12, 2.25)
81	0.61	0.74	1.72	9.03	0.04	(1.18, 2.09)
82	0.55	0.61	2.28	12.19	0.04	(1.02, 2.32)
83	0.67	0.83	2.13	11.23	0.04	(1.10, 2.16)
84	0.48	0.46	1.94	10.32	0.05	(0.96, 1.38)
85	0.62	0.77	1.72	9.05	0.04	(1.18, 2.09)
86	0.54	0.59	2.47	13.39	0.04	(1.02, 2.22)
87	0.68	0.84	2.13	11.26	0.04	(1.08, 2.16)
88	0.49	0.48	1.94	10.33	0.05	(0.95, 1.37)
89	0.59	0.68	1.53	8.07	0.05	(1.16, 2.19)
90	0.55	0.61	1.72	9.01	0.04	(1.12, 2.11)
91	0.64	0.80	2.28	12.15	0.04	(1.05, 2.28)
92	0.47	0.44	1.53	8.10	0.05	(1.12, 2.25)
93	0.61	0.74	1.72	9.03	0.04	(1.18, 2.09)
94	0.56	0.63	1.94	10.30	0.04	(0.99, 1.33)
95	0.41	0.39	2.08	11.15	0.04	(0.94, 1.32)
96	0.58	0.57	1.72	9.05	0.03	(0.78, 1.21)
97	0.51	0.54	2.47	13.41	0.05	(1.05, 2.32)
98	0.69	0.85	2.13	11.29	0.03	(1.12, 2.08)
99	0.45	0.42	1.94	10.31	0.04	(0.98, 1.29)
100	0.63	0.76	1.72	9.03	0.04	(1.21, 2.09)
101	0.68	0.84	2.13	11.26	0.04	(1.08, 2.16)
102	0.49	0.48	1.94	10.33	0.05	(0.95, 1.37)
103	0.59	0.68	1.53	8.07	0.05	(1.16, 2.19)
104	0.55	0.61	1.72	9.01	0.04	(1.12, 2.11)
105	0.64	0.80	2.28	12.15	0.04	(1.05, 2.28)
106	0.47	0.44	1.53	8.10	0.05	(1.12, 2.25)
107	0.61	0.74	1.72	9.03	0.04	(1.18, 2.09)
108	0.56	0.63	1.94	10.30	0.04	(0.99, 1.33)
109	0.41	0.39	2.08	11.15	0.04	(0.94, 1.32)
110	0.58	0.57	1.72	9.05	0.03	(0.78, 1.21)
111	0.51	0.54	2.47	13.41	0.05	(1.05, 2.32)
112	0.69	0.85	2.13	11.29	0.03	(1.12, 2.08)
113	0.45	0.42	1.94	10.31	0.04	(0.98, 1.29)
114	0.63	0.76	1.72	9.03	0.04	(1.21, 2.09)
115	0.54	0.59	2.47	13.39	0.04	(1.02, 2.22)
116	0.68	0.84	2.13	11.26	0.04	(1.08, 2.16)
117	0.49	0.48	1.94	10.33	0.05	(0.95, 1.37)
118	0.59	0.68	1.53	8.07	0.05	(1.16, 2.19)
119	0.55	0.61	1.72	9.01	0.04	(1.12, 2.11)
120	0.64	0.80	2.28	12.15	0.04	(1.05, 2.28)
121	0.47	0.44	1.53	8.10	0.05	(1.12, 2.25)
122	0.61	0.74	1.72	9.03	0.04	(1.18, 2.09)
123	0.56	0.63	1.94	10.30	0.04	(0.99, 1.33)
124	0.41	0.39	2.08	11.15	0.04	(0.94, 1.32)
125	0.58	0.57	1.72	9.05	0.03	(0.78, 1.21)
126	0.51	0.54	2.47	13.41	0.05	(1.05, 2.32)
127	0.69	0.85	2.13	11.29	0.03	(1.12, 2.08)
128	0.45	0.42	1.94	10.31	0.04	(0.98, 1.29)
129	0.63	0.76	1.72	9.03	0.04	(1.21, 2.09)
130	0.54	0.59	2.47	13.39	0.04	(1.02, 2.22)
131	0.68	0.84	2.13	11.26	0.04	(1.08, 2.16)
132	0.49	0.48	1.94	10.33	0.05	(0.95, 1.37)
133	0.59	0.68	1.53	8.07	0.05	(1.16, 2.19)
134	0.55	0.61	1.72	9.01	0.04	(1.12, 2.11)
135	0.64	0.80	2.28	12.15	0.04	(1.05, 2.28)
136	0.47	0.44	1.53	8.10	0.05	(1.12, 2.25)
137	0.61	0.74	1.72	9.03	0.04	(1.18, 2.09)
138	0.56	0.63	1.94	10.30	0.04	(0.99, 1.33)
139	0.41	0.39	2.08	11.15	0.04	(0.94, 1.32)
140	0.58	0.57	1.72	9.05	0.03	(0.78, 1.21)
141	0.51	0.54	2.47	13.41	0.05	(1.05, 2.32)
142	0.69	0.85	2.13	11.29	0.03	(1.12, 2.08)
143	0.45	0.42	1.94	10.31	0.04	(0.98, 1.29)
144	0.63	0.76	1.72	9.03	0.04	(1.21, 2.09)
145	0.54	0.59	2.47	13.39	0.04	(1.02, 2.22)
146	0.68	0.84	2.13	11.26	0.04	(1.08, 2.16)
147	0.49	0.48	1.94	10.33	0.05	(0.95, 1.37)
148	0.59	0.68	1.53	8.07	0.05	(1.16, 2.19)
149	0.55	0.61	1.72	9.01	0.04	(1.12, 2.11)
150	0.64	0.80	2.28	12.15	0.04	(1.05, 2.28)
151	0.47	0.44	1.53	8.10	0.05	(1.12, 2.25)
152	0.61	0.74	1.72	9.03	0.04	(1.18, 2.09)
153	0.56	0.63	1.94	10.30	0.04	(0.99, 1.33)
154	0.41	0.39	2.08	11.15	0.04	(0.94, 1.32)
155	0.58	0.57	1.72	9.05	0.03	(0.78, 1.21)
156	0.51	0.54	2.47	13.41	0.05	(1.05, 2.32)
157	0.69	0.85	2.13	11.29	0.03	(1.12, 2.08)
158	0.45	0.42	1.94	10.31	0.04	(0.98, 1.29)
159	0.63	0.76	1.72	9.03	0.04	(1.21, 2.09)
160	0.54	0.59	2.47	13.39	0.04	(1.02, 2.22)
161	0.68	0.84	2.13	11.26	0.04	(1.08, 2.16)
162	0.49	0.48	1.94	10.33	0.05	(0.95, 1.37)
163	0.59	0.68	1.53	8.07	0.05	(1.16, 2.19)
164	0.55	0.61	1.72	9.01	0.04	(1.12, 2.11)
165	0.64	0.80	2.28	12.15	0.04	(1.05, 2.28)
166	0.47	0.44	1.53	8.10	0.05	(1.12, 2.25)
167	0.61	0.74	1.72	9.03	0.04	(1.18, 2.09)
168	0.56	0.63	1.94	10.30	0.04	(0.99, 1.33)
169	0.41	0.39	2.08	11.15	0.04	(0.94, 1.32)
170	0.58	0.57	1.72	9.05	0.03	(0.78, 1.21)
171	0.51	0.54	2.47	13.41	0.05	(1.05, 2.32)
172	0.69	0.85	2.13	11.29	0.03	(1.12, 2.08)
173	0.45	0.42	1.94	10.31	0.04	(0.98, 1.29)
174	0.63	0.76	1.72	9.03	0.04	(1.21, 2.09)
175	0.54	0.59	2.47	13.39	0.04	(1.02, 2.22)
176	0.68	0.84	2.13	11.26	0.04	(1.08, 2.16)
177	0.49	0.48	1.94	10.33	0.05	(0.95, 1.37)
178	0.59	0.68	1.53	8.07	0.05	(1.16, 2.19)
179	0.55	0.61	1.72	9.01	0.04	(1.12, 2.11)
180	0.64	0.80	2.28	12.15	0.04	(1.05, 2.28)
181	0.47	0.44	1.53	8.10	0.05	(1.12, 2.25)
182	0.61	0.74	1.72	9.03	0.04	(1.18, 2.09)
183	0.56	0.63	1.94	10.30	0.04	(0.99, 1.33)
184	0.41	0.39	2.08	11.15	0.04	(0.94, 1.32)
185	0.58	0.57	1.72	9.05	0.03	(0.78, 1.21)
186	0.51	0.54	2.47	13.41	0.05	(1.05, 2.32)
187	0.69	0.85	2.13	11.29	0.03	(1.12, 2.08)
188	0.45	0.42	1.94	10.31	0.04	(0.98, 1.29)
189	0.63	0.76	1.72	9.03	0.04	(1.21, 2.09)
190	0.54	0.59	2.47	13.39	0.04	(1.02, 2.22)
191	0.68	0.84	2.13	11.26	0.04	(1.08, 2.16)
192	0.49	0.48	1.94	10.33	0.05	(0.95, 1.37)
193	0.59	0.68	1.53	8.07	0.05	(1.16, 2.19)
194	0.55	0.61	1.72	9.01	0.04	(1.12, 2.11)
195	0.64	0.80	2.28	12.15	0.04	(1.05, 2.28)
196	0.47	0.44	1.53	8.10	0.05	(1.12, 2.25)
197	0.61	0.74	1.72	9.03	0.04	(1.18, 2.09)
198	0.56	0.63	1.94	10.30	0.04	(0.99, 1.33)
199	0.41	0.39	2.08	11.15	0.04	(0.94, 1.32)
200	0.58	0.57	1.72	9.05	0.03	(0.78, 1.21)
201	0.51	0.54	2.47	13.41	0.05	(1.05, 2.32)
202	0.69	0.85	2.13	11.29	0.03	(1.12, 2.08)
203	0.45	0.42	1.94	10.31	0.04	(0.98, 1.29)
204	0.63	0.76	1.72	9.03	0.04	(1.21, 2.09)
205	0.54	0.59	2.47	13.39	0.04	(1.02, 2.22)
206	0.68	0.84	2.13	11.26	0.04	(1.08, 2.16)
207	0.49	0.48	1.94	10.33	0.05	(0.95, 1.37)
208	0.59	0.68	1.53	8.07	0.05	(1.16, 2.19)
209	0.55	0.61	1.72	9.01	0.04	(1.12, 2.11)
210	0.64	0.80	2.28	12.15	0.04	(1.05, 2.28)
211	0.47	0.44	1.53	8.10	0.05	(1.12, 2.25)
212	0.61	0.74	1.72	9.03	0.04	(1.18, 2.09)
213	0.56	0.63	1.94	10.30	0.04	(0.99, 1.33)
214	0.41	0.39	2.08	11.15	0.04	(0.94, 1.32)
215	0.58	0.57	1.72	9.05	0.03	(0.78, 1.21)
216	0.51	0.54	2.47	13.41	0.05	(1.05, 2.32)
217	0.69	0.85	2.13	11.29	0.03	(1.12, 2.08)
218	0.45	0.42	1.94	10.31	0.04	(0.98, 1.29)
219	0.63	0.76	1.72	9.03	0.04	(1.21, 2.09)
220	0.54	0.59	2.47	13.39	0.04	(1.02, 2.22)
221	0.68	0.84	2.13	11.26	0.04	(1.08, 2.16)
222	0.49	0.48	1.94	10.33	0.05	(0.95, 1.37)
223	0.59	0.68	1.53	8.07	0.05	(1.16, 2.19)
224	0.55	0.61	1.72	9.01	0.04	(1.12, 2.11)
225	0.64	0.80	2.28	12.15	0.04	(1.05, 2.28)
226	0.47	0.44	1.53	8.10	0.05	(1.12, 2.25)
227	0.61	0.74	1.72	9.03	0.04	(1.18, 2.09)
228	0.56	0.63	1.94	10.30	0.04	(0.99, 1.33)
229	0.41	0.39	2.08	11.15	0.04	(0.94, 1.32)
230	0.58	0.57	1.72	9.05	0.03	(0.78, 1.21)
231	0.51	0.54	2.47	13.41	0.05	(1.05, 2.32)
232	0.69	0.85	2.13	11.29	0.03	(1.12, 2.08)
233	0.45	0.42	1.94	10.31	0.04	(0.98, 1.29)
234	0.63	0.76	1.72	9.03	0.04	(1.21, 2.09)
235	0.54	0.59	2.47	13.39	0.04	(1.02, 2.22)
236	0.68	0.84	2.13	11.26	0.04	(1.08, 2.16)
237	0.49	0.48	1.94	10.33	0.05	(0.95, 1.37)
238	0.59	0.68	1.53	8.07	0.05	(1.16, 2.19)
239	0.55	0.61	1.72	9.01	0.04	(1.12, 2.11)
240	0.33	0.35	2.45	13.12	0.05	(0.85, 1.07)

As indicated by the results, HUD interface layout design can be adjusted to assist cognitive judgment and decision-making and optimize the capture and processing of attention to driving task information. The unreasonable AR-HUD visual design will exceed the visual capacity limit of the driver while increasing the risk of driving accidents. The experimental results suggested that as the perception task involved in AR-HUD visual search tasks was increased, participants’ attention to the driving scene in front declined, the visual search range was narrowed, and the scanning path length was shortened. In a limited time, participants should fully focus on the information of the entire driving scene. The scanning path length was significantly different from that of non-driving tasks, probably due to the different positions and distributions of environmental elements that attract participants’ visual attention, which may reduce the effectiveness of scanning paths (43–47). In other words, the layout of AR-HUD visual elements may affect visual scanning strategies, such that drivers’ cognitive load can be affected.

### 3.3. Implementation of BP-GA

#### 3.3.1. An algorithm for integrating genetic algorithm and BP neural network

**Figure 5:**
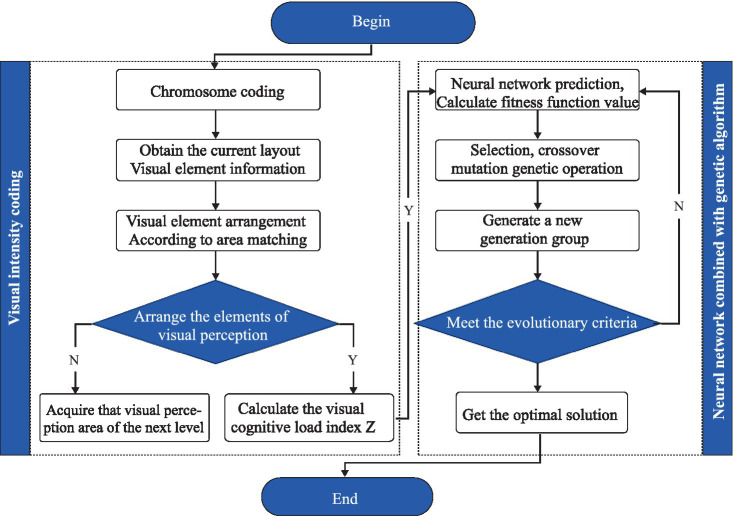
Calculation process of AR-HUD visual cognitive load prediction model.

Recent research has employed a fitness function developed by a BP neural network and genetic algorithm to optimize the interface design of AR-HUD interactive systems based on driver’s visual distribution characteristics, yielding effective results (48). In this study, a combination of machine learning and deep learning was adopted to compare optimization effects. The BP neural network model, using AR-HUD visual interaction element coding and visual cognitive load index as input and output layers, was incorporated into a genetic algorithm to determine the optimal AR-HUD design (49). Figure 5 illustrates the relevant process.

#### 3.3.2. Topological structure of BP neural network

**Figure 6:**
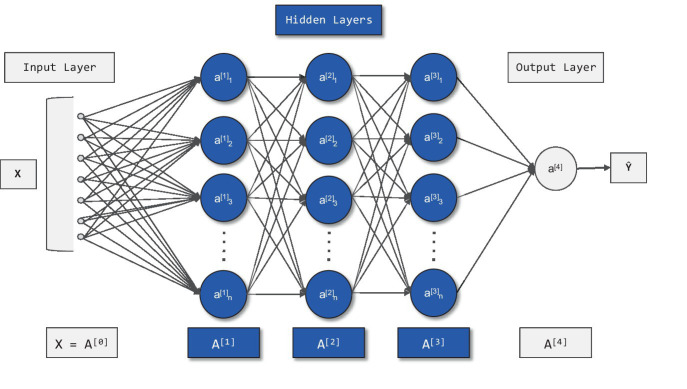
Topological structure of BP neural network.

According to practical issues, the topological structure of neural network is determined, including three layers: 1 input layer, 3 hidden layer and 1 output layer. The number of neurons in the input layer is 26 and the number of neurons in the output layer is 1. In the hidden layer, the optimal number of neurons is determined by heuristic method (50, 51), and the optimal number of neurons is determined to be 15 after operation. Lastly, based on the BP neural network between the visual arrangement coding of the head-up display and NASA-TLX, its topological structure is determined, as shown in Figure 6. The meaning of a^[1]^_(x)_ represents the activation function of the hidden layer in the backpropagation (BP) neural network. A^[0]^ represents the input layer, and A^[1]^-A^[3]^ represent the hidden layers. A^[4]^ represents the output layer. X represents the input variable, and Y represents the output variable.

#### 3.3.3. Performance of BP neural network method

The activation function of neurons refers to an integral part of BP neural networks, which should be differentiable, and its derivative should be continuous (48, 52). Thus, the log-sigmoid activation function {logsig} was selected as the activation function for the hidden layer and output layer neurons of the BP neural network. The {Levenberg–Marquardt} BP algorithm training function {trainlm} was used in the training process of the BP network modeling. The maximum iteration number of the neural network was 1000 times, the training target error was set to 10, the learning rate was 0.1, and the status was displayed every five training cycles. Unmodified parameters served as the default values of the system (53, 54). The neural network fitting toolbox ran in Matlab software. After repeated training and weight adjustment, the optimal validation performance index was 0.349 at epoch 6.

#### 3.3.4. Chromosome coding of visual cognitive load model

As depicted in Table 2, this study’s AR-HUD visual model comprised nine discrete variables, i.e., GM, GL, GF, GA, GB, GC, GD, GE, and GF, which had been encoded as 26-bit binary strings in the previous neural network model construction. For instance, the 26-bit binary string of the design scheme in Figure 6 corresponds to 1000010000011001010100110, in which the respective binary character represents a gene. Among the above-mentioned variables, GM, GL, GF, and GA served as 4-bit binary variables, whereas the acted as are 2-bit binary variables. In the integrated operation of neural network and genetic algorithm, the chromosome input in binary coding form was first converted to floating-point type, and then the floating-point value was converted back to the identical binary form as the input after the calculation of the adaptive function. The code conversion rules are presented as follows: a 4-bit binary code was converted to a floating-point number that continuously ranges from 0 to 4. For continuous variables with values of [0,1], [1,2], [2,3], and [3,4], the corresponding binary codes turned out to be 1000, 0100, 0010, and 0001, respectively. A two-bit binary code was converted to a floating-point number in the range of [0,2]. For continuous variables with values of [0,1] and [1,2], the corresponding binary codes reached 10 and 01 (55), respectively. The approximate optimal solution can only be explained after decoding.

**Table 2:** Chromosome coding of visual cognitive load model.

Element	Content	Chromosome coding and interpretation			
GM	Main color	RGB:#2979FF	RGB:#FE0000	RGB:#4ADE80	RGB:#F26D21
		1000	0100	0010	0001
GL	Arrangement	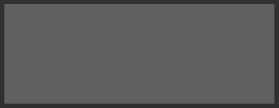	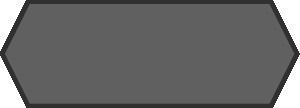	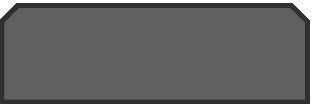	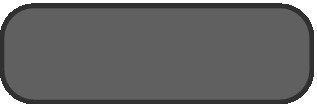
		1000	0100	0010	0001
GF	Frame shape	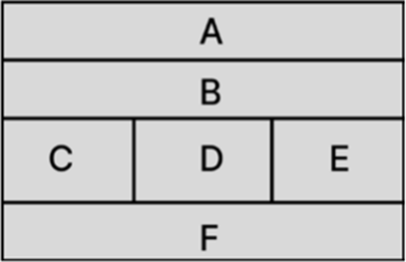	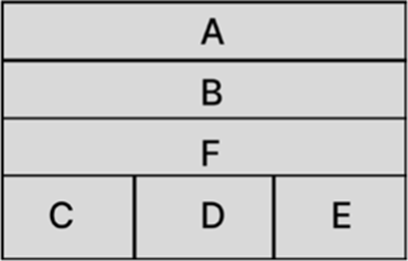	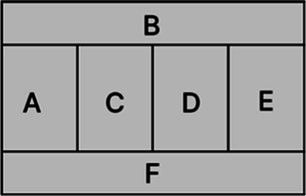	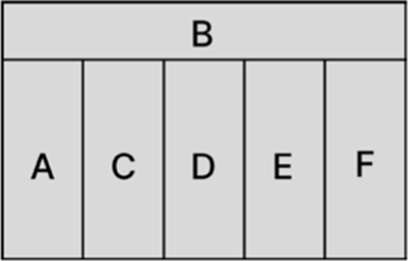
		1000	0100	0010	0001
GA: Module A	Information:	Roboto	Avenir Next	Burlingame	Tipperary
		1000	0100	0010	0001
GB: Module B	Navigation	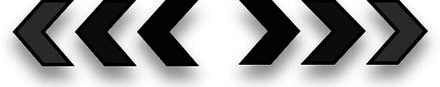		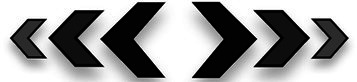	
GC: Module C	Speed Indicator	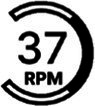		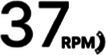	
		10		01	
GD: Module D	Speed table	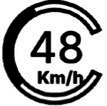		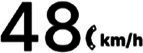	
		10		01	
GE: Module E	Driving state	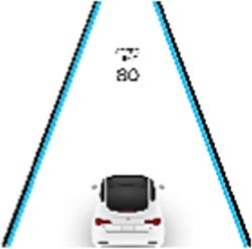		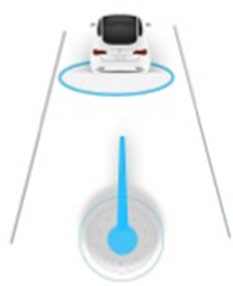	
		10		01	
GF: Module F	Gear status	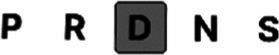		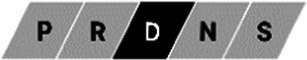	
		10		01	

To establish a neural network prediction model, this study constructed a training sample set. The input is chromosome coding of AR-HUD visual model, and the output is the average NASA-TLX of the 240 AR-HUD prototypes rated by the user in the experiment. After organizing the data, the input and output data of the neural network model were determined, as shown in Table 3.

**Table 3:** Input and output of neural network prediction model.

Number	Chromosomal code	NASA-TLX
1	00101000010010000101011001	12.10
2	00100100010001001001100110	12.60
3	00100010101000010010011001	5.52
4	00100001100100100010100110	4.25
5	00011000010001010010010110	6.93
6	00010100010010100010101001	9.71
7	00010010100100000101010110	11.42
8	00010001101000001001101001	11.14
9	00010001101001001001101011	9.16
10	00010001101101001001101011	13.12
11	00010100010001001001100110	9.13
12	00010001100100100010100100	11.33
13	10000100010010101000101001	14.32
14	01000001000100100010100100	6.82
15	01000010100100001001010110	5.52
16	00100100010010100010101001	8.13
17	01000010100100000101010110	12.13
18	10000001101000001001101001	8.12
19	00010001101001001001101010	10.32
20	01100101111010101100110000	10.30
21	10101001011110101100110001	12.20
22	11011011011101110101100011	9.40
23	01010111001111010101010010	7.90
24	11101010101001011010100001	6.80
25	00101010111010110101100011	8.60
26	10101010101011010101100011	11.50
27	01110101011011010101010001	13.20
28	11101101010101011010100001	6.10
29	01010101010110110101010011	9.90
30	11010101011010110101100001	7.50
31	00101010101010110101100001	11.80
32	10101010101010101101010011	8.30
33	01101010101011010101010010	14.10
34	11110101010101011010100010	6.50
35	01010101010101010101100010	10.60
36	11010101010101110101010010	12.60
37	00101010101010110101010011	9.20
38	10101010101010110101010000	7.70
39	01101010101010110101010011	13.70
40	11101010101010101101010010	6.90
41	01010101010101010101010011	11.10
42	11010101010101010101100011	8.90
43	00101010101010110101010010	6.70
44	10101010101010110101010001	9.80
45	01101010101010110101010010	12.10
46	11101010101010101010100010	7.10
47	01010101010101010101010000	10.90
48	11010101010101010101010000	8.10
49	00101010101010110101010011	11.60
50	10101010101010110101010001	6.30
51	01101010101010110101010001	13.90
52	11101010101010101010100000	6.60
53	01010101010101010101010010	10.10
54	11010101010101010101010001	12.40
55	00101010101010110101010001	9.50
56	10101010101010110101010010	7.60
57	01101010101010110101010000	14.00
58	11101010101010101010100011	6.20
59	01010101010101010101010001	11.00
60	11010101010101010101010010	9.70
61	00101010101010110101010011	7.30
62	10101010101010110101010000	12.00
63	01101010101010110101010010	8.70
64	11101010101010101010100010	6.40
65	01010101010101010101010001	10.70
66	11010101010101010101010011	12.90
67	00101010101010110101010000	8.80
68	10101010101010110101010011	7.10
69	01101010101010110101010000	14.00
70	11101010101010101010100011	6.30
71	01010101010101010101010010	10.20
72	11010101010101010101010001	12.60
73	00101010101010110101010001	9.20
74	10101010101010110101010011	7.70
75	01101010101010110101010011	13.70
76	11101010101010101010100010	6.90
77	01010101010101010101010010	10.30
78	11010101010101010101010001	12.20
79	00101010101010110101010011	9.40
80	10101010101010110101010000	7.90
81	01101010101010110101010011	14.10
82	11101010101010101010100000	6.10
83	01010101010101010101010000	10.00
84	11010101010101010101010010	12.50
85	00101010101010110101010001	9.30
86	10101010101010110101010000	7.80
87	01101010101010110101010011	13.80
88	11101010101010101010100010	6.50
89	01010101010101010101010010	10.60
90	11010101010101010101010001	12.90
91	00101010101010110101010000	8.80
92	10101010101010110101010001	7.10
93	01101010101010110101010000	14.00
94	11101010101010101010100011	6.20
95	01010101010101010101010001	10.20
96	11010101010101010101010011	12.60
97	00101010101010110101010010	9.50
98	10101010101010110101010001	7.60
99	01101010101010110101010011	13.70
100	11101010101010101010100010	6.70
101	01010101010101010101010010	10.90
102	11010101010101010101010010	8.10
103	00101010101010110101010011	11.60
104	10101010101010110101010001	6.30
105	01101010101010110101010001	13.90
106	11101010101010101010100001	6.60
107	01010101010101010101010010	10.10
108	11010101010101010101010001	12.40
109	00101010101010110101010001	9.50
110	10101010101010110101010000	7.60
111	01101010101010110101010000	14.00
112	11101010101010101010100011	6.20
113	01010101010101010101010001	11.50
114	11010101010101010101010001	12.30
115	00101010101010110101010001	9.70
116	10101010101010110101010011	7.30
117	01101010101010110101010010	12.80
118	11101010101010101010100010	6.40
119	01010101010101010101010001	10.70
120	11010101010101010101010011	12.90
121	00101010101010110101010000	8.80
122	10101010101010110101010011	7.10
123	01101010101010110101010000	14.00
124	11101010101010101010100011	6.30
125	01010101010101010101010010	10.20
126	11010101010101010101010001	12.60
127	00101010101010110101010001	9.20
128	10101010101010110101010011	7.70
129	01101010101010110101010011	13.70
130	11101010101010101010100010	6.90
131	01010101010101010101010010	10.30
132	11010101010101010101010001	12.20
133	00101010101010110101010011	9.40
134	10101010101010110101010000	7.90
135	01101010101010110101010011	14.10
136	11101010101010101010100000	6.10
137	01010101010101010101010000	10.00
138	11010101010101010101010010	12.50
139	00101010101010110101010001	9.30
140	10101010101010110101010000	7.80
141	01101010101010110101010011	13.80
142	11101010101010101010100010	6.50
143	01010101010101010101010010	10.60
144	11010101010101010101010001	12.90
145	00101010101010110101010000	8.80
146	10101010101010110101010001	7.10
147	01101010101010110101010000	14.00
148	11101010101010101010100011	6.20
149	01010101010101010101010001	10.20
150	11010101010101010101010011	12.60
151	00101010101010110101010010	9.50
152	10101010101010110101010001	7.60
153	01101010101010110101010011	13.70
154	11101010101010101010100010	6.70
155	01010101010101010101010010	10.90
156	11010101010101010101010010	8.10
157	00101010101010110101010011	11.60
158	10101010101010110101010001	6.30
159	01101010101010110101010001	13.90
160	11101010101010101010100000	6.60
161	01010101010101010101010010	10.10
162	11010101010101010101010001	12.40
163	00101010101010110101010001	9.50
164	10101010101010110101010000	7.60
165	01101010101010110101010011	14.00
166	11101010101010101010100011	6.20
167	01010101010101010101010001	11.50
168	11010101010101010101010001	12.30
169	00101010101010110101010001	9.70
170	10101010101010110101010011	7.30
171	01101010101010110101010010	12.80
172	11101010101010101010100010	6.40
173	01010101010101010101010001	10.70
174	11010101010101010101010011	12.90
175	00101010101010110101010000	8.80
176	10101010101010110101010001	7.10
177	01101010101010110101010000	14.00
178	11101010101010101010100011	6.30
179	01010101010101010101010010	10.20
180	11010101010101010101010001	12.60
181	00101010101010110101010001	9.20
182	10101010101010110101010011	7.70
183	01101010101010110101010011	13.70
184	11101010101010101010100010	6.90
185	01010101010101010101010010	10.30
186	11010101010101010101010001	12.20
187	00101010101010110101010011	9.40
188	10101010101010110101010000	7.90
189	01101010101010110101010011	14.10
190	11101010101010101010100000	6.10
191	01010101010101010101010000	10.00
192	11010101010101010101010010	12.50
193	00101010101010110101010001	9.30
194	10101010101010110101010000	7.80
195	01101010101010110101010011	13.80
196	11101010101010101010100010	6.50
197	01010101010101010101010010	10.60
198	11010101010101010101010001	12.90
199	00101010101010110101010000	8.80
200	10101010101010110101010001	7.10
201	01101010101010110101010000	14.00
202	11101010101010101010100011	6.20
203	01010101010101010101010001	11.00
204	11010101010101010101010010	9.70
205	00101010101010110101010011	7.30
206	10101010101010110101010000	12.30
207	01101010101010110101010001	8.70
208	11101010101010101010100010	6.40
209	01010101010101010101010001	10.70
210	11010101010101010101010011	12.90
211	00101010101010110101010000	8.80
212	10101010101010110101010001	7.10
213	01101010101010110101010000	14.00
214	11101010101010101010100011	6.20
215	01010101010101010101010001	11.50
216	11010101010101010101010001	12.30
217	00101010101010110101010001	9.70
218	10101010101010110101010011	7.30
219	01101010101010110101010010	12.80
220	11101010101010101010100010	6.40
221	01010101010101010101010001	10.70
222	11010101010101010101010011	12.90
223	00101010101010110101010000	8.80
224	10101010101010110101010001	7.10
225	01101010101010110101010000	14.00
226	11101010101010101010100011	6.20
227	01010101010101010101010001	11.50
228	11010101010101010101010001	12.30
229	00101010101010110101010001	9.70
230	10101010101010110101010011	7.30
231	01101010101010110101010010	12.80
232	11101010101010101010100010	6.40
233	01010101010101010101010001	10.70
234	11010101010101010101010011	12.90
235	00101010101010110101010000	8.80
236	10101010101010110101010001	7.10
237	01101010101010110101010000	14.00
238	11101010101010101010100011	6.20
239	01010101010101010101010001	11.50
240	10000010101101001001100011	13.12

#### 3.3.5. Parameters of IVPM-GA

The initial population size was the total number of samples in the AR-HUD dataset 240. The mutation rates range of GA algorithm in this study was from 0.01 (1%) to 0.1 (10%). The crossover rate of GA reached 0.8 (80%). Roulette wheel selection method was adopted to select individuals from the population for reproduction (12).

### 3.4. Implementation of IVPM-GA

A deep learning-powered image view point prediction model (IVPM) was introduced in this study to improve AR-HUDs (Augmented Reality Head-Up Displays) design with a strong focus on occupational safety and health (OSH). Based on Bylinskii et al.’s work (50), the IVPM, trained with human visual attention data, can be conducive to optimizing retargeting and thumbnails design. The model was further integrated into a design tool providing real-time feedback. Its application in AR-HUD design aimed at lessening cognitive load and enhancing driver safety, emphasizing the effective communication of critical information, such that its significance in promoting OSH can be highlighted.

#### 3.4.1. Dataset of interface designs

IVPM uses the eye movement experimental dataset in section 3.1 for training, which contains 240 ground truth (GT) significance markers developed by AR-HUD HCI, and divides the training set and the test set according to 80–20%.

#### 3.4.2. The loss function and model architecture of IVPM

The equation of importance for IVPM to predict the content of each pixel position in a bitmap image is shown in Eq. (1). The importance prediction Pi∈[0,1] is output for each pixel, with higher values suggesting greater importance. Similar to saliency models that perform well on natural images, IVPM is based on the FCN architecture. Given the true importance Qi∈[0,1] at each pixel i, the sigmoid cross-entropy loss is optimized over all pixels = 1, 2, …, N to optimize the FCN model parameters θ (50):

(1) $$L\left(\theta \right)=-\frac{1}{N}\overset{N}{\underset{i=1}{\sum }}\left(Q_{i}\log P_{i}+\left(1-Q_{i}\right)\log\left(1-P_{i}\right)\right)$$

Where P_i = σ(f_i(θ)) represents the importance prediction value obtained by passing the FCN output f(θ) through the sigmoid activation function σ(X) = (1 + exp(−x))-1. Notably, this loss function has been commonly employed for binary classification, given that Q_i∈{0,1}. To be specific, it is extended to real-valued Q_i∈[0,1].

**Figure 7:**
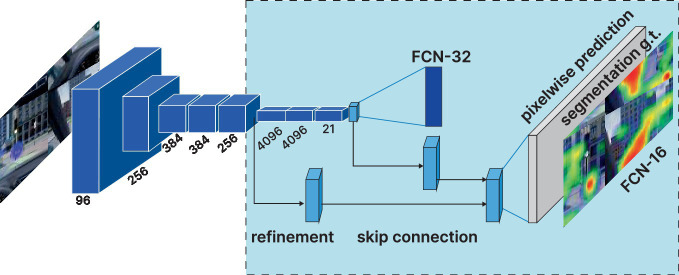
Image viewpoint prediction model (IVPM) architecture: fully participatory networks can efficiently learn to make dense predictions for per-pixel tasks like semantic segmentation.

After continuous pooling, the model prediction turned out to be 1/32 of the input image resolution. To improve the prediction resolution and capture finer details, skip connections from earlier layers were introduced, following the steps proposed by Long et al. to form the FCN-16 s model (56, 57). As indicated by the experimental results, FCN-16 s (with skip connections from pool4) captured more details and improved prediction performance compared with the FCN-32 s model (due to limited samples, the pre-trained FCN-32 s model was used to initialize the network parameters and fine-tuned). The model architecture is shown in Figure 7 (58).

## 4. Results

### 4.1. Performance of IVPM model method

Kullback–Leibler divergence ( KL ) and cross correlation ( CC ) are used to assess the similarity between the forecast map and the GT importance marker map. KL severely punishes the wrong prediction, so the sparse graph that fails to predict the important position of GT will get a higher KL value (low score). Given the GT importance graph q and the predicted importance graph p, the P,KL value is calculated as Eq. (2) (48):

(2) $$KL\left(P,Q\right)=\overset{N}{\underset{i=1}{\sum }}\left(Q_{i}\log Q_{i}-Q_{i}\log P_{i}\right)=L\left(P,Q\right)-H\left(Q\right)$$

In addition, H(Q) = − l( $Q_{i}\log Q_{i}$ ) is the entropy of the importance graph of GT, and L(P,Q) is the cross entropy of the predicted value and GT. A large KL divergence indicates a large difference between graphs, and KL(P,Q) =0 indicates that two graphs are the same. CC is calculated as Eq. (3) (48):

(3) $$CC\left(P,Q\right)=\frac{\frac{1}{N}\sum_{i=1}^{N}\left(P_{i}-P\right)\left(Q_{i}-Q\right)}{\sqrt{\frac{1}{N}\sum_{i=1}^{N}{\left(P_{i}-P\right)}^{2}}\sqrt{\frac{1}{N}\sum_{i=1}^{N}{\left(Q_{i}-Q\right)}^{2}}}$$

**Figure 8:**
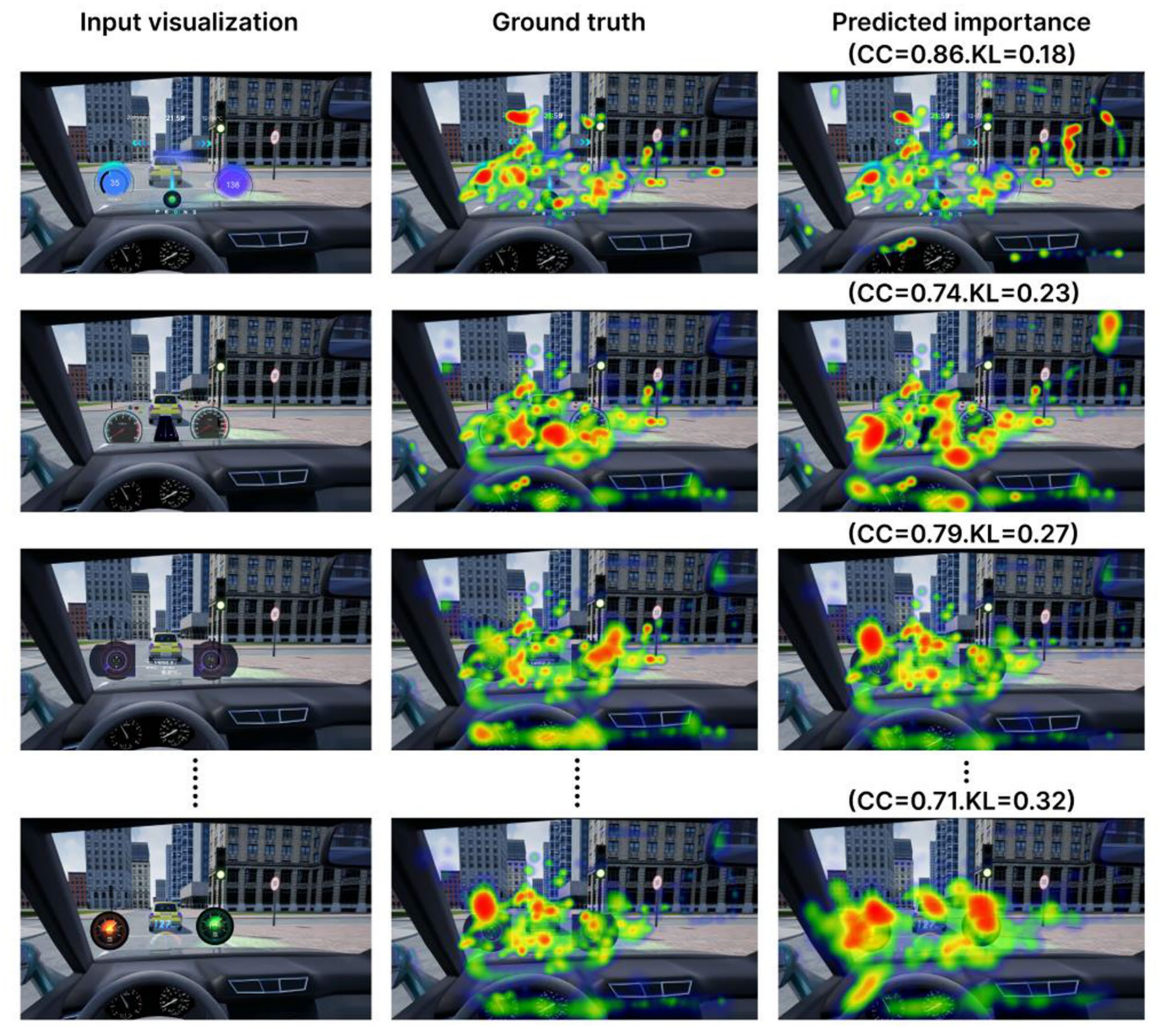
Importance predictions for Eye tracking hotspot data visualizations, compared with ground truth View and sorted by performance. The model is good at localizing Eye tracking hotspot visualization as well as picking up the extreme points on graphs.

Where $P=\frac{1}{N}\overset{N}{\underset{i=1}{\sum }}P_{i}$ , and q is the same. CC ranges from −1 to 1; CC equal to 1 suggests that the greatest correlation exists between graphs P and Q ; high CC score and low KL score suggest that the good prediction effect. In the experiment on the test image dataset, the average score of CC was 0.715, KL and the average score of KL reached 0.313, such that good performance prediction was achieved. Only some results are presented in Figure 8.

### 4.2. BP-GA operation result

**Figure 9:**
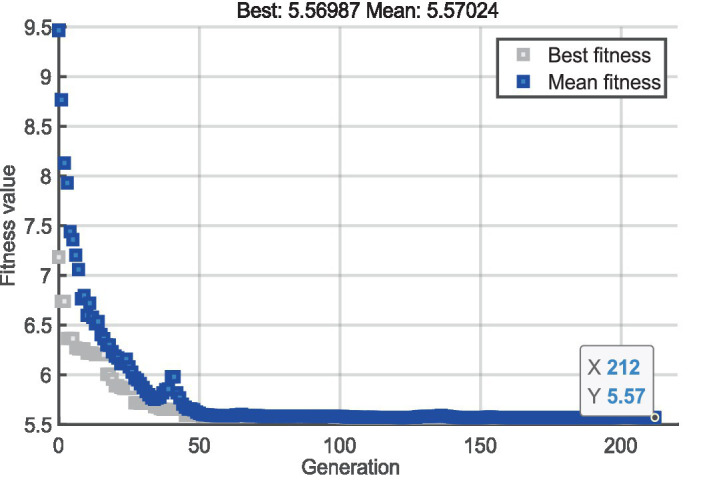
Genetic algorithm fitness evolution process diagram.

Taking the BP neural network function of cognitive load prediction established in section 4.1 as the fitness function of genetic algorithm, a genetic algorithm is established. By adjusting parameters and running the program multiple times, as shown in Figure 9, the evolution process is plotted with the generation number on the *x*-axis and the maximum fitness of individuals on the *y*-axis. The design model in the initial randomly generated parent population has a relatively high quality, which to some extent avoids the phenomenon of local convergence in the optimization process. The maximum fitness of individual samples gradually increases with the iteration of the population. After 212 iterations, the optimal individual is found and its preference is approximately 5.570.

### 4.3. IVPM-GA operation result

**Figure 10:**
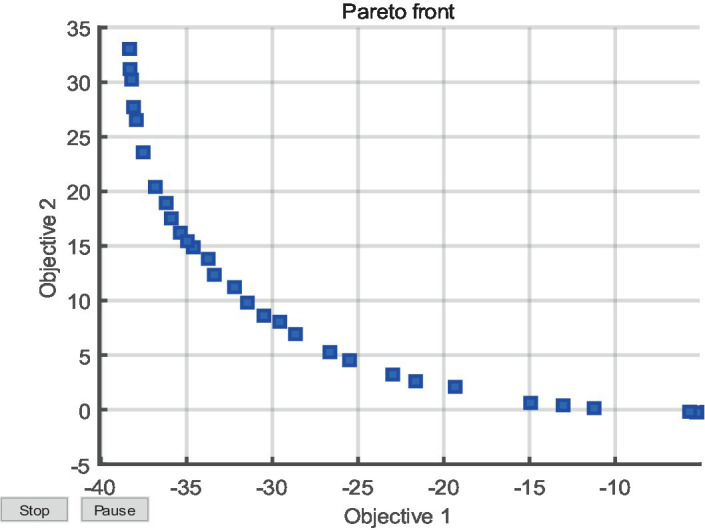
Evolution process of multi-objective optimization Pareto Front based on IVPM genetic algorithm.

A genetic algorithm fitness function is established by combining the predicted NASA-TLX value and the IVPM function into a single fitness function. The function is developed to consider the minimum prediction error between the actual and predicted NASA-TLX minimum value and the predicted image viewpoint as multi-objective optimization indicators. The fitness function is used in a multi-objective optimization algorithm based on genetic algorithm, in which the distribution is updated once per generation as the algorithm evolves. After 40 iterations of the gene population, the optimal individual is identified. Upon the termination of the iteration, the individual distribution graph depicted in Figure 10 is obtained.

### 4.4. Comparison of results using the CH scale

The genetic algorithm was employed to obtain the optimal encoding rule for the AR-HUD interface design problem. After decoding was completed, two optimized interfaces were obtained: one using the IVPM method and the other based on the BP method. The cognitive loads of the two solutions were assessed by comparing IVPM-GA and BP-GA.

The Cooper-Harper (CH) rating scale, which subjectively evaluates driving difficulty on a scale of ten levels, was used for comparison. A total of 15 participants assessed their driving experience and perceptions of the difficulty levels (59–64). The collected rating scale data was adopted to calculate the average scores of corresponding factors and total scores for the respective solution, and the corresponding load level strengths were examined. Table 4 lists the relevant results.

**Table 4:** Comparison of grade distribution of driving performance assessment IVPM-GA and BP-GA with CH scale.

IVPM-GA > BP-GA		IVPM-GA = BP-GA		IVPM-GA < BP-GA	
Grade	*n*	Grade	*n*	Grade	*n*
5/4	2	4/4	1	4/5	0
6/4	1	5/5	1	5/6	1
6/5	2	6/6	0	6/7	1
7/5	2	7/7	1		
7/6	4				
Sum	11		3		1

The results in the table indicate that 11 out of 15 participants (73.3% of the total) rated the IVPM-GA solution higher than the BP-GA solution. As indicated by the above result, the AR-HUD optimized using IVPM-GA outperforms the one optimized using only BP-GA in terms of the driving performance, such that the effectiveness of the algorithm adopted in this study is confirmed.

## 5. Discussion

### 5.1. Interpretation of the results

As revealed by the results of this study, the AR-HUD interface optimized using the IVPM-GA method outperformed the BP-GA method in terms of the driving performance. The genetic algorithm based on the IVPM method was also found to be effective in optimizing the interface design. Furthermore, the Cooper-Harper rating scale results suggested that the IVPM-GA method was preferred by the majority of the participants.

In terms of occupational health and safety, the findings of this study are significant as they provide evidence that the IVPM-GA method can enhance driving performance and user experience. The above finding is vital to professional drivers who face occupational health and safety risks (e.g., fatigue and cognitive load). By reducing cognitive load and improving driving performance, AR-HUD interfaces optimized using the IVPM-GA method can contribute to reducing occupational health and safety risks for professional drivers.

### 5.2. Implications of the findings

The results of the study not only confirmed the effectiveness of the genetic algorithm optimization methods for AR-HUD interface design while taking on great significance in the safety and health of professional drivers. The optimized AR-HUD interface using the IVPM method significantly improved driving performance compared with the BP-GA method. This finding suggests that optimized AR-HUD interfaces can potentially improve driver safety on the road. Moreover, the Cooper-Harper rating scale results indicated that the IVPM method was preferred by the majority of the participants, suggesting that the optimized interface could improve user experience and reduce visual fatigue, which can benefit the occupational health of professional drivers.

### 5.3. Limitations and future directions for research

In brief, future research on real-time image processing for driving applications should focus on performance and efficiency while consider the implications of OSH. It is imperative to assess the cognitive load, distractions, and overall effect on driver well-being. Furthermore, optimizing the system’s user interface and integrating it with driver assistance systems can further improve OSH in driving scenarios. By addressing the above-described aspects, researchers can develop real-time image processing systems that enhance driving performance and occupational safety and health. The implications of real-time image processing for Occupational Safety and Health (OSH) in driving scenarios should be considered (65). As the suggested network operates in real time and is employed in driving scenarios, it exerts direct effects on driver safety and well-being (66). Subsequent research should assess OSH effects of real-time image processing in driving scenarios (56), which comprise cognitive load and potential distractions (57, 67). Quantitative measures (e.g., eye-tracking and physiological monitoring) provide insights into the mental workload and attention demands (68). Besides, a focus should be placed on mitigating potential negative OSH effects (69). It is imperative to optimize the user interface to reduce cognitive load and distractions. (70). Principles (e.g., efficient information layout and intuitive design) are capable of ensuring the system enhances performance while reducing adverse effects (60, 71). Integrating real-time image processing with driver assistance systems can improve OSH in driving scenarios. (72). Accordingly, cognitive load can be reduced, and overall driving safety can be improved (73).

## 6. Conclusion

### 6.1. Summary of the research findings

This study confirmed the superiority of the IVPM-GA method over the conventional BP-GA method for classical HCI optimization design under AR-HUD interface optimization for professional drivers. The IVPM-based genetic algorithm is effective in optimizing the interface design while improving driving performance and enhancing user experience. The above-mentioned results take on vital significance in the safety and well-being of professional drivers since optimized AR-HUD interfaces are promising in lowering cognitive load, reducing visual distractions, and ultimately enhancing driver safety.

### 6.2. Final thoughts and future research directions

The importance and necessity of this study lie in its potential to dramatically enhance the occupational health and safety (OHS) of professional drivers. By utilizing machine learning to predict cognitive load in AR-HUD design, this study can lay a solid basis for significantly enhancing driving performance, user experience, and above all, the occupational health of drivers, an often overlooked yet critically important facet of professional driving. The emphasis of this study on driver OHS was the need to reduce fatigue, mitigate risks of accidents, and improve overall health conditions, primarily through the optimization of visual ergonomics and the reduction of cognitive loads. The potential benefits are vast, such that the day-to-day experiences of professional drivers can be affected, and longer-term health outcomes and safety records can be facilitated.

Future research inspired by this study could delve into the application of a broad range of machine learning methods and optimization techniques. It is imperative to expand sample sizes and engage diverse participant groups, such that the findings can be ensured to be generalizable and applicable across a wide spectrum of professional drivers. An important direction for future research is exploring how optimized AR-HUD interfaces can integrate with existing driver assistance systems, providing timely, relevant information and further contributing to the reduction of cognitive load, ultimately enhancing overall driving safety.

The results of this study are critical to inform future Occupational Safety and Health (OSH) policies and training programs, offering an evidence-based method to interface design that centers on driver safety and wellness. This study represents a significant contribution to the expansive field of human-computer interaction, emphasizing the beneficial integration of advanced technologies (e.g., AR-HUD). It elucidates how the above-described technologies can be efficiently adopted to enhance driver safety and occupational health. Accordingly, this study advocates for the development of safer and more ergonomic professional driving environments, a requisite consideration in the modern fast-paced world, substantiating its relevance to OSH.

## Data availability statement

The original contributions presented in the study are included in the article/supplementary material, further inquiries can be directed to the corresponding author.

## Ethics statement

The studies involving human participants were reviewed and approved by the Ethics Committee of Lingnan Normal University. The patients/participants provided their written informed consent to participate in this study. Written informed consent was obtained from the individual(s) for the publication of any potentially identifiable images or data included in this article.

## Author contributions

JT: conceptualization, methodology, validation, visualization, and writing—original draft. FW: funding acquisition. YK: software and resources. J-KK: conceptualization, supervision, and project administration. All authors contributed to the article and approved the submitted version.

## Funding

This study was funded by Experimental Management Research Fund of Guangdong Higher Education Society (GDJ 2016037); Industry-University-Research Innovation Fund of Science and Technology Development Center of Ministry of Education (2018C01051). 2023 Lingnan Normal University Education and Teaching Reform Project.

## Conflict of interest

The authors declare that the research was conducted in the absence of any commercial or financial relationships that could be construed as a potential conflict of interest.

## Publisher’s note

All claims expressed in this article are solely those of the authors and do not necessarily represent those of their affiliated organizations, or those of the publisher, the editors and the reviewers. Any product that may be evaluated in this article, or claim that may be made by its manufacturer, is not guaranteed or endorsed by the publisher.
